# New Appliances for Hospital Use

**Published:** 1907-10-05

**Authors:** 


### NEW APPLIANCES FOR HOSPITAL USE.

### MESSRS. DAVIS BENNETT AND CO.'S SANITARY APPLIANCES.

Messrs. Davis Bennett and Co., of Horseferry Road, London, S.W., are well known as makers of apparatus suitable for hospital use, the dominant note of which is practical utility. Their water-closets are all made with large areas, and some of them with jets at back and front, so that all solid excreta are easily carried off. Forms of closets are made by the firm so arranged that the excreta can only fall into the centre of the water space, in place of, as in so many cases, being lodged upon the back of the pan. Other forms are made with specially low seats, and with water cushions fitted to them, the seats being so arranged that the user can lift himself up by projections provided in front. For out-patients' departments a closet is made which gives a slight flush, automatically, when sat upon, and a heavy flush, also automatically, on rising. The firm have also arranged a ventilator for the sanitary block of the hospital, worked by the flushing water. The water is led to a small tank above the usual flush tank, and in running down works a small turbine wheel, which operates a fan, passing 300 cubic feet of air out of the closet with each charge of water. There are baths for lunatics, made by the firm, so arranged that scalding is not easily accomplished.

In lavatories for operating theatres the firm makes a very useful contribution to the methods of controlling the water supply. Two levers, for hot and cold water, are fixed a few inches from the floor, similar to the elbow levers described in the articles dealing with this subject. The levers are easily moved by the foot, and remain in any position until again moved, so that the surgeon can manipulate them without balancing himself, and wishing he had three feet. The waste is opened by an elbow lever.

Messrs. Davis Bennett and Co. make a special form of opal tile, which, being only fired once, instead of twice, they claim does not craze. They are also bringing out a new material for the manufacture of baths, lavatories, and sanitary appliances generally, which they have named King's ware. It is composed of fireclay, flint, and porcelain, in certain proportions, and is stated to be less porous, and more durable than simple fireclay. They make the usual line of sinks and wash-up closets, with special features of their own, which are claimed to add to the efficiency of the apparatus, and they also make several fittings for drains, enabling them to be swept from the surface without an inspection chamber. The firm's showrooms are very well laid out, and will well repay a visit from anyone who has to spend any money on fitting up a hospital.

### NEW ENGINEERING APPLIANCES.

A fitting of some interest in institutions where large steam laundries are in use has recently been applied in several Metropolitan hospitals for the purpose of rendering only one steam main working at high pressure necessary, instead of one high pressure and one low pressure main. The low-pressure piping is generally used for supplying steam to the washing machines and boiling vessels, etc., because at high pressure the steam would have too great a tendency to boil the water out of the compartments, thus causing a nuisance and considerable waste both of steam and water, besides being possibly dangerous. In the arrangement now adopted, however, the steam piping to these vessels is taken directly off the high-pressure main, and a throttling device, capable of accurate regulation, is situated immediately against the valve controlling the steam to each of the various units, so that even if the valve is opened fully only a certain flow of steam can be passed capable of meeting the maximum demands efficiently and no more. The result is very satisfactory, and saves loss of steam and part of the cost of upkeep. The fitting is not patented, and can be obtained at present from Messrs. T. Potter and Sons, Kirby Street, Hatton Garden, E.C., and Messrs. J. and F. May, 33-35 Whetstone Park, Lincoln's Inn Fields, W.C. The prices are about 10s. each for ¾-inch diameter size and 7s. 6d. for ½-inch.

